# Management and outcomes in a consecutive series of patients with aero-digestive fistula at a tertiary gastro-esophageal surgery center

**DOI:** 10.1093/dote/doad068

**Published:** 2023-12-14

**Authors:** Fahad Murad, Fredrik Klevebro, Gert Henriksson, Ioannis Rouvelas, Mats Lindblad, Magnus Nilsson

**Affiliations:** Division of Surgery and Oncology, Department of Clinical Science Intervention and Technology (CLINTEC) Karolinska Institutet, and Department of Upper Abdominal Diseases, Karolinska University Hospital, Stockholm, Sweden; Division of Surgery and Oncology, Department of Clinical Science Intervention and Technology (CLINTEC) Karolinska Institutet, and Department of Upper Abdominal Diseases, Karolinska University Hospital, Stockholm, Sweden; Division of Ear, Nose and Throat Diseases, Department of Clinical Science Intervention and Technology (CLINTEC) Karolinska Institutet, and Department of Ear, Nose and Throat Diseases, Karolinska University Hospital, Stockholm, Sweden; Division of Surgery and Oncology, Department of Clinical Science Intervention and Technology (CLINTEC) Karolinska Institutet, and Department of Upper Abdominal Diseases, Karolinska University Hospital, Stockholm, Sweden; Division of Surgery and Oncology, Department of Clinical Science Intervention and Technology (CLINTEC) Karolinska Institutet, and Department of Upper Abdominal Diseases, Karolinska University Hospital, Stockholm, Sweden; Division of Surgery and Oncology, Department of Clinical Science Intervention and Technology (CLINTEC) Karolinska Institutet, and Department of Upper Abdominal Diseases, Karolinska University Hospital, Stockholm, Sweden

**Keywords:** aero-digestive fistula, covered metal stent, definitive surgical repair, latissimus dorsi muscle-flap

## Abstract

Aerodigestive fistula (ADF) is defined as a pathological connection between the upper digestive tract and the airway. ADF is associated with high morbidity and mortality and management is often complex. A cohort study including all patients admitted with ADF 2004–2022 at a single tertiary esophageal surgery center was performed based on prospectively collected administrative data and retrospectively collected electronic patient chart data,. Patient demographics, performance status, comorbidity, fistula characteristics, management, and outcomes in terms of morbidity and mortality were assessed in patients with ADF of three distinct types: (i) tumor overgrowth-related, (ii) various benign etiologies, and (iii) post-esophagectomy. Sixty-one patients with ADF were included in the study, 33 (54.1%) tumor overgrowth-related, six (9.8%) benign and 22 (36.1%) post-esophagectomy. In the post-esophagectomy group 15 out of 22 (68.2%) patients were diagnosed with anastomotic leakage prior to ADF diagnosis. Self-expandable metallic stents (SEMS) were used for temporary fistula sealing in 59 out of 61 (96.7%) patients, of which most received stents in both the digestive tract and airway. Temporary fistula sealing with stents was successful enabling discharge from hospital in 47 out of 59 (79.7%) patients. Definitive ADF repair was performed in 16 (26.2%) patients, of which one (6.3%) died within 90-days and 15 could be discharged home with permanently sealed fistulas. ADF is a complex condition associated with high mortality, which often requires multiple advanced interventions. SEMS can be applied in the airway and simultaneously in the digestive tract to temporarily seal the ADF as bridge to definitive surgical repair.

## INTRODUCTION

Aerodigestive fistula (ADF) is defined as a pathological connection between the upper digestive tract, usually the esophagus or a conduit replacing the esophagus, and the airway. An ADF can involve the trachea, main bronchi, or less commonly more distal segmental bronchi. ^1^ ADF can be congenital or acquired, of which this study only addresses the acquired type. A common type of acquired ADF is due to tumor overgrowth with tumor necrosis leading to fistulation, either by esophageal cancer affecting the airways, or occasionally the inverse by overgrowth of airway malignancies or mediastinal tumors into the esophagus.^2^ Tumor overgrowth ADFs are particularly common in the context of response to oncological treatment with chemotherapy and/or radiotherapy. Acquired ADF can also be caused by ingestion of caustic compounds, trauma, infections, for example tuberculosis, long term tracheostomy use, pressure necrosis from esophageal stenting, or post esophagectomy, caused by an anastomotic leakage or surgical trauma.

The most typical clinical sign of an ADF is coughing when drinking, as fluid passes into the airway. ADF causes several clinical problems, most importantly related to contamination of gastrointestinal content into the airway causing bronchopulmonary inflammatory response and pneumonia. Mortality is high, especially in fistulas occurring due to tumor overgrowth.^3^ ADF can be diagnosed by computerized tomography (CT) with water soluble oral contrast and should be confirmed with bronchoscopy and upper gastrointestinal endoscopy (UGE). During UGE (with carbon dioxide insufflation) increased levels carbon dioxide returned to the ventilator (end-tidal CO_2_) can usually be detected, which is a pathognomonic sign of ADF.^4^

Treatment of ADF has historically been upfront surgical repair, often using muscle flaps and other autologous patching tissues as well as synthetic or biological off-the-shelf materials.^5^ As ADF patients often have a combination of pneumonitis with severe inflammatory response and pneumonia in the acute phase and consequently seriously affecting outcomes from upfront major surgery, many centers have sought minimally invasive solutions aiming to seal the ADF. Different endoscopic techniques have been tried, including biological glue techniques, endoscopic clipping, stenting techniques and the Amplatzer device.^3^^,^^6^^,^^7^ A well-documented way of minimally invasive sealing of the fistula is using covered self-expandable metallic stents (SEMS) both in the alimentary tract and airway, so-called double stenting.^3^^,^^5^ However, the overall experience as reflected in the literature is that these techniques very rarely seal ADF permanently.^1^^,^^3^^,^^6^^,^^8^^,^^9^ Definitive surgical ADF repair can be performed with or without esophageal or gastric conduit resection. A muscle flap from the latissimus dorsi placed between the esophagus or gastric conduit and airway is one technique for sealing the ADF that can be applied with successful outcomes.^9^

In our institution we have developed a management algorithm, using the damage-control concept from trauma surgery, in which we first aim to temporarily seal the fistula with covered SEMS in both the alimentary tract and airway. When the patient has returned home and recovered to a good performance status definitive surgery is performed electively. We have previously published the results of our ADF management in two articles in 2009 and 2013.^10^^,^^11^

This study represents an unselected consecutive single institutional case series at a tertiary gastro-esophageal surgery center comprising all patients with ADF managed during a 19-year period. The aim of the study is to describe the causes, management, and outcomes of this group of patients during the study period.

## METHODS

### Design and patients

**Fig. 1:**
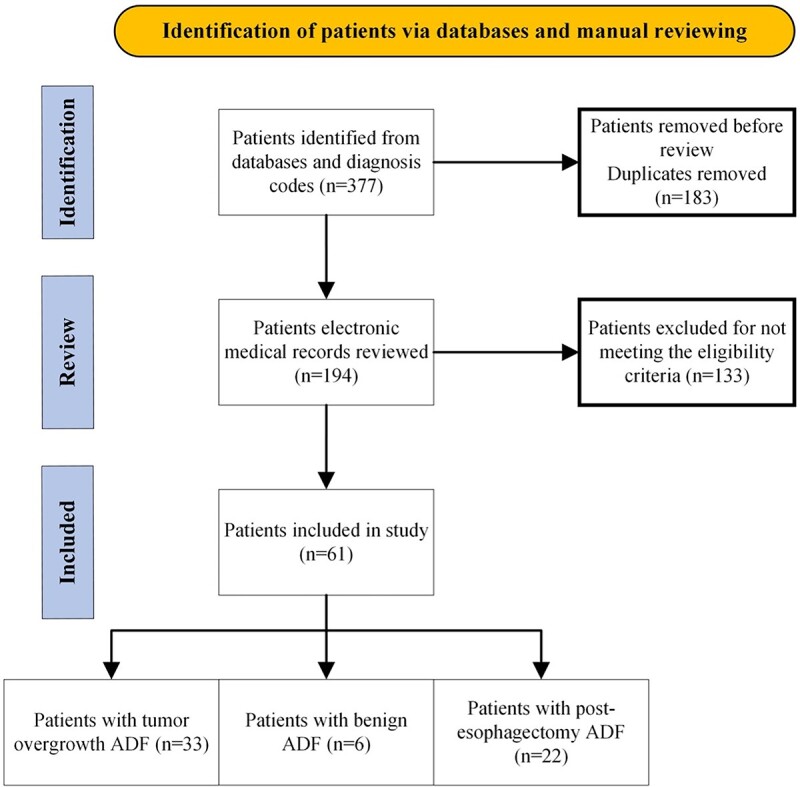
Flow chart describing the inclusion of patients in the study.

A retrospective cohort study based on prospectively collected data. All patients presenting at or referred to the Karolinska University Hospital, a tertiary gastro-esophageal surgery referral center for ADF in Scandinavia, between 2004 and 2022 were included. Patients were identified from the institutional database by retrieving all patients admitted to the Department of Upper Abdominal Diseases that underwent an airway procedure either diagnostic or interventional procedure codes. These included flexible or rigid bronchoscopy with or without biopsy from the trachea, endoscopic placement of stent in airways, other surgery, or endoscopic surgery on trachea. Patients’ electronic medical records were then reviewed, duplicates removed and patients not meeting the inclusion criteria were excluded. Data included patient characteristics, treatment and surgical details, histopathology, and survival. Patients were divided into three groups based on the etiology of ADF, including: (i) tumor overgrowth-related, (ii) various benign etiologies, for example ingestion of caustic compounds, and (iii) post-esophagectomy as shown in Figure 1.

### Outcomes

The primary outcome was survival from the time of ADF diagnosis. Secondary outcomes included mortality after definitive surgery, total length of hospital stay, total ICU stay, successful temporary sealing of ADF defined as discharge to home and overall success of definitive treatment of ADF, based on the 30- and 90-day mortality and 1-year survival post operatively.

### Ethical aspects

The study was conducted in accordance with the Helsinki Declaration and ethical approval was granted by regional and national medical ethics authorities (2018/970-31/1, 2022-02634-02).

### Statistical analysis

Categorical variables were reported as absolute numbers and percentages. Normally distributed continuous variables were reported as mean ± standard deviation (SD) and non-normally distributed variables as median and range. Kaplan–Meier graphs were used to demonstrate overall and progression-free survival. Statistical calculations were made using Stata IC 16.1 software.

## RESULTS

A total of 61 patients were included in the study, representing the total number of patients with ADF presenting at or being referred to the Karolinska University Hospital tertiary gastro-esophageal surgery center between 2004 and 2022. Among these, 33 (54.1%) had developed a tumor overgrowth ADF, 6 (9.8%) an ADF due to benign non-surgical causes and 22 patients (36.1%) developed an ADF post-esophagectomy. Median age for all patients was 66 years, and 67 for the tumor overgrowth ADF group, 61 years for the benign ADF group and 66 for the post esophagectomy group. Forty-seven (77.1%) patients were male, and 14 (22.9%) female. Patient baseline characteristics and disease-related demographics were similar in the tumor overgrowth ADF and post-esophagectomy ADF groups as shown in Table 1. Forty-six patients (75.4%) had a Charlson comorbidity index of >4, and the majority of patients ,59/61), (96.7%) had an American Society of Anesthesiologists (ASA) score of ≥2, and 43 (70.5%) patients had a history of smoking (Table 1).

**Table 1:** Baseline characteristics of all aero-digestive fistula (ADF) patients

*n (%)*		Post-esophagectomy ADF	Tumor overgrowth ADF	Benign ADF	Total
Total		22	33	6	61
Age, median (range)		67 (58–78)	66 (20–78)	61 (50–78)	66 (20–78)
Gender					
	Male	20 (90.9)	22 (66.7)	5 (83.3)	47 (77.1)
	Female	2 (9.1)	11 (33.3)	1 (16.7)	14 (22.9)
Charlson comorbidity index					
	0–2	0 (0)	1 (3.0)	0 (0)	1 (1.6)
	3–4	4 (18.2)	4 (12.1)	0 (0)	8 (13.1)
	>4	18 (81.8)	26 (78.8)	2 (33.3)	46 (75.4)
	Missing	0	2	4	6
ASA score					
	0	1 (4.6)	0 (0)	0 (0)	1 (1.6)
	I	1 (4.6)	0 (0)	0 (0)	1 (1.6)
	II	7 (31.8)	12 (36.3)	3 (50.0)	22 (36.1)
	III	7 (31.8)	19 (57.6)	3 (50.0)	29 (47.5)
	IV	6 (27.3)	2 (6.1)	0 (0)	8 (13.1)
ECOG Performance status*					
	0	7 (31.8)	5 (15.2)	0 (0)	12 (19.7)
	1	2 (9.1)	9 (27.3)	2 (33.3)	13 (21.3)
	2	1 (4.6)	14 (42.4)	3 (50.0)	18 (29.5)
	3	5 (22.7)	3 (9.1)	0 (0)	8 (13.1)
	4	7 (31.8)	2 (6.1)	1 (16.7)	10 (16.4)
Smoking history					
	Yes	16 (72.7)	24 (72.7)	3 (50.0)	43 (70.5)
	No	5 (22.7)	5 (15.2)	2 (33.3)	12 (19.7)
	Missing	1	4	1	6

^*^ *At earliest presentation*

In the tumor overgrowth ADF group most patients had locally advanced esophageal cancer with 23 (69.7%) patients staged as clinical T4 and only six (18.2%) patients had no metastases to regional lymph nodes. Seven (21.2%) patients had stage 4 disease with distant metastases. Six of these patients (18.2%) had a primary tumor in the proximal third of the esophagus whilst 16 (48.5%) had a tumor in the middle third, four (12.1%) had a tumor in the distal third, one (3%1) in the gastroesophageal junction, and six (18.2%) with non-esophageal cancer. Concerning treatment, four patients (12.1%) received palliative chemotherapy and five (15.2%) neoadjuvant chemotherapy. In 17 (51.5%) patients with tumor overgrowth ADF presented during or after definitive chemotherapy, while seven patients presented with tumor overgrowth ADF without any prior tumor-directed treatment (Table 2).

**Table 2:** Tumor characteristics and tumor directed treatment in patients with tumor overgrowth related aero-digestive fistulas (ADF)

*n (%)*		
Clinical tumor stage		
	T1	0 (0)
	T2	1 (3.0)
	T3	6 (18.2)
	T4	23 (69.7)
	Missing	3
Clinical nodal stage		
	N0	6 (18.2)
	N1	9 (27.3)
	N2	8 (24.2)
	N3	7 (21.2)
	Missing	3
Distant metastasis		
	Yes	7 (21.2)
	No	23 (69.7)
	Missing	3
Primary tumor location		
	Proximal	6 (18.2)
	Middle	16 (48.5)
	Distal	4 (12.1)
	Gastroesophageal junction	1 (3.0)
	Non esophageal cancer	6 (18.2)
	Missing	0
Histology		
	Esophageal squamous cell carcinoma	22 (66.7)
	Esophageal adenocarcinoma	3 (9.1)
	Small cell lung carcinoma	1 (3.0)
	Neuroendocrine tumor	1 (3.0)
	Mesothelioma	1 (3.0)
	Lymphoma	1 (3.0)
	Missing	4
Tumor directed treatment		
	Neoadjuvant chemotherapy	0 (0)
	Palliative chemotherapy	4 (12.1)
	Neoadjuvant chemoradiotherapy	5 (15.2)
	Definitive chemoradiotherapy	17 (51.5)
	None	7 (21.2)
	Missing	0

The benign etiology group, in which all patients in this study had an ADF caused by ingestion of caustic compounds, (Table 1) had a median age of 61 years (range 50–78) with five (83.3%) male patients. Two patients (33.3%) had a Charlson comorbidity index >2 and all six patients (100%) had an ASA-score of ≥2. Two (33.3%) patients had Eastern cooperative oncology group (ECOG) performance status 1, three (50%) had a score of 2 and one patient (16.7%) had a score of 4. Three (50%) had a history of smoking.

In the post-esophagectomy group, most patients had undergone a resection for a distal tumor, 10 (45.5%) in the distal third of the esophagus, seven (31.8%) in the gastroesophageal junction and five (22.7%) in the middle third of the esophagus. Of these, 14 (63.6%) patients had adenocarcinomas, and seven (31.8%) had squamous cell carcinomas. Concerning tumor-directed treatment, eight (36.3%) patients had undergone esophageal resection only, while four (18.2%) had received neoadjuvant chemotherapy and 10 (45.5%) neoadjuvant chemoradiotherapy. Post-esophagectomy ADF occurred after a known anastomotic leakage in 15/22 (68.2%). The anastomotic leakages were treated with esophageal stents in five patients and endoscopic vacuum therapy using the EsoSponge^12^ in five patients, prior to ADF diagnosis (Table 3).

**Table 3:** Patient characteristics and treatment details before fistula presentation in patients with post-esophagectomy aero-digestive fistulas (ADF)

*N (%)*		Post-esophagectomy ADF (*n* = 22)
Clinical tumor stage		
	T1	4 (18.2)
	T2	1 (4.6)
	T3	17 (77.3)
	T4	0 (0)
Clinical nodal stage		
	N0	11 (50.0)
	N1	8 (36.4)
	N2	3 (13.6)
	N3	0 (0)
Tumor location		
	Proximal	0 (0)
	Middle	5 (22.7)
	Distal	10 (45.5)
	Gastroesophageal junction	7 (31.8)
Histology		
	Esophageal squamous cell carcinoma	7 (31.8)
	Esophageal adenocarcinoma	14 (63.6)
	Missing	1
Tumor directed treatment		
	Esophageal resection only	8 (36.3)
	Neoadjuvant chemotherapy	4 (18.2)
	Neoadjuvant chemoradiotherapy	10 (45.5)
	Missing	0
**Anastomotic leakage diagnosed before ADF presentation, including treatment**		
Known anastomotic leakage before ADF presentation		
	Yes	15 (68.2)
	No	7 (31.8)
	Missing	0
Number of esophageal stents per patient used for anastomotic leakage treatment		
	1	3 (13.6)
	2–4	2 (9.1)
	>4	0 (0)
	None	17 (77.3)
	Missing	0
Number of EsoSponges used per patient used for anastomotic leakage treatment		
	1–2	4 (18.1)
	>2	1 (4.6)
	None	17 (77.3)
	Missing	0
Total number of days with EsoSponge		
	0–10	1 (4.6)
	>10	4 (18.1)
	None	17 (77.3)
	Missing	0

Thirty-seven (60.7%) patients had ADF to the trachea, 13 patients (21.3%) had ADF to the left main bronchus. ADF from all etiology-groups occurred most often in the trachea, but main bronchial fistula in the tumor overgrowth ADF group was most common on the left side while right main bronchial fistula was more common in post-esophagectomy ADF group. Acute treatment with both airway and esophageal SEMS were used in 56 (91.8%) patients, one patient (1.6%) received an airway SEMS only, two patients (3.3%) received esophageal SEMS only, and two (3.3%) patients received no acute endoscopic or surgical treatment. Temporary fistula sealing with SEMS was successful in 47 out of 59 (79.7%) patients in total, 24 out of 32 (75%) in the tumor-overgrowth group, four out of five (80%) patients in the benign ADF group and 19 out of 22 (86.4%) in the post-esophagectomy group. Types and number of SEMS placed is shown in Table 4.

**Table 4:** Aero-digestive fistula (ADF) characteristics and management, excluding definite surgical management, by etiology of ADF

*n (%)*		Post-esophagectomy ADF	Tumor overgrowth ADF	Benign ADF	Total
Location of ADF					
	Trachea	13 (59.1)	19 (57.6)	5 (83.3)	37 (60.7)
	Left main bronchus	2 (9.1)	10 (30.3)	1 (16.7)	13 (21.3)
	Right main bronchus	5 (22.7)	1 (3.0)	0 (0)	6 (9.8)
	Distal bronchi	2 (9.1)	3 (9.1)	0 (0)	5 (8.2)
	Missing	0	0	0	0
Acute treatment of ADF					
	Airway stent only	1 (4.6)	0 (0)	0 (0)	1 (1.6)
	Esophageal stent only	0 (0)	2 (6.1)	0 (0)	2 (3.3)
	Airway and esophageal stenting	21 (95.4)	30 (91.0)	5 (83.3)	56 (91.8)
	None	0 (0)	1 (3.0)	1 (16.7)	2 (3.3)
	Missing	0	0	0	0
Total number of esophageal stents placed after ADF presentation					
	1	10 (45.5)	19 (57.6)	2 (33.3)	31 (50.8)
	2–4	6 (27.3)	13 (39.4)	2 (33.3)	21 (34.4)
	>4	5 (22.7)	0 (0)	1 (16.7)	6 (9.8)
	None	1 (4.6)	1 (3.0)	1 (16.7)	3 (4.9)
	Missing	0	0	0	0
Total number of airway stents placed after ADF presentation					
	1	11 (50.0)	19 (57.6)	1 (16.7)	31 (50.8)
	2–4	9 (40.9)	9 (27.3)	4 (66.7)	22 (36.1)
	>4	2 (9.1)	2 (6.1)	0 (0)	4 (6.6)
	None	0 (0)	3 (9.1)	1 (16.7)	4 (6.6)
	Missing	0	0	0	0
Successful temporary sealing of fistula with stents					
Total		22	32	5	59
	Yes	19 (86.4)	24 (75.0)	4 (80.0)	47 (79.7)
	No	3 (13.6)	8 (25.0)	1 (20.0)	12 (20.3)

In total 16 (26.2%) patients were treated with definitive surgical repair according to the management model previously described. The delay from ADF diagnosis to definitive treatment ranged from 2 to 234 days. Of these 16 patients, three (18.8%) were in the tumor overgrowth ADF group, four (25%) in the benign fistula group and nine (56.3%) in the post-esophagectomy group. Of the 16 operated patients 11 (68.8%) underwent fistula repair with latissimus dorsi muscle flap. One patient (6.3%) died within 90 days of definitive surgery, (Table 5). After definitive surgery, one (33.3%) patient survived 1 year after surgery in the tumor overgrowth ADF group, two (50%) in the benign fistula group, and six (66.7%) in the post-esophagectomy ADF group. In total nine (56.3%) patients that underwent definitive surgery survived 1 year.

**Table 5:** Description of subgroup undergoing definitive surgical management of aero-digestive fistula (ADF), including mortality outcomes

*n (%)*	Post-esophagectomy ADF	Tumor overgrowth ADF	Benign ADF	Total
Total	9	3	4	16
Repair of fistula with latissimus dorsi muscle flap				
Yes	6 (66.7)	3 (100)	2 (50.0)	11 (68.8)
No	3 (33.3)	0 (0)	2 (50.0)	5 (31.2)
30-day mortality after definitive treatment	1 (11.1)	0 (0)	0 (0)	1 (6.3)
90-day mortality after definitive treatment	1 (11.1)	0 (0)	0 (0)	1 (6.3)
One-year survival	6 (66.7)	1 (33.3)	2 (50.0)	9 (56.3)

In the acute phase treatment 31 (50.8%) patients in total required ICU-stay. Of these 20 (32.8%) required a 1–10-day ICU-stay, seven (11.5%) a 10–20-day ICU-stay and four (6.6%) a > 20-day ICU-admission (Table 6). Nineteen patients (31.1%) survived one year or more after fistula diagnosis. Seven (11.5%) survived 3 years or more and three patients (4.9%) survived 5 years or more (Table 6).

**Table 6:** Description of outcomes of all aerodigestive fistula patients (intention-to-treat population)

*n (%)*		Post-esophagectomy ADF	Tumor overgrowth ADF	Benign ADF	Total
30-day mortality		3 (13.6)	9 (27.3)	0	12 (19.7)
90-day mortality		4 (18.2)	15 (45.5)	1 (16.7)	20 (32.8)
1-year survival		13 (59.1)	4 (12.1)	2 (33.3)	19 (31.1)
3-year survival		5 (22.7)	1 (3.0)	1 (16.7)	7 (11.5)
5-year survival		2 (9.1)	0 (0)	1 (16.7)	3 (4.9)
ICU stay (days)					
	0	7 (31.8)	22 (66.7)	1 (16.7)	30 (49.2)
	1–10	9 (40.9)	8 (24.2)	3 (50.0)	20 (32.8)
	10–20	5 (22.7)	2 (6.1)	0 (0)	7 (11.5)
	>20	1 (4.6)	1 (3.0)	2 (33.3)	4 (6.6)
	Missing	0	0	0	0

**Fig. 2:**
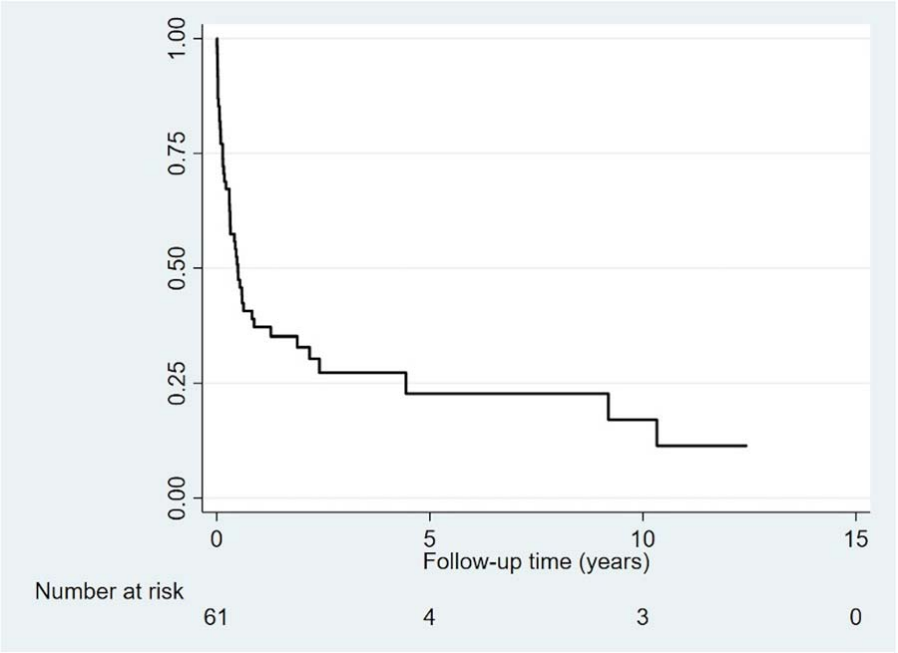
Overall survival in the whole study cohort.

**Fig. 3:**
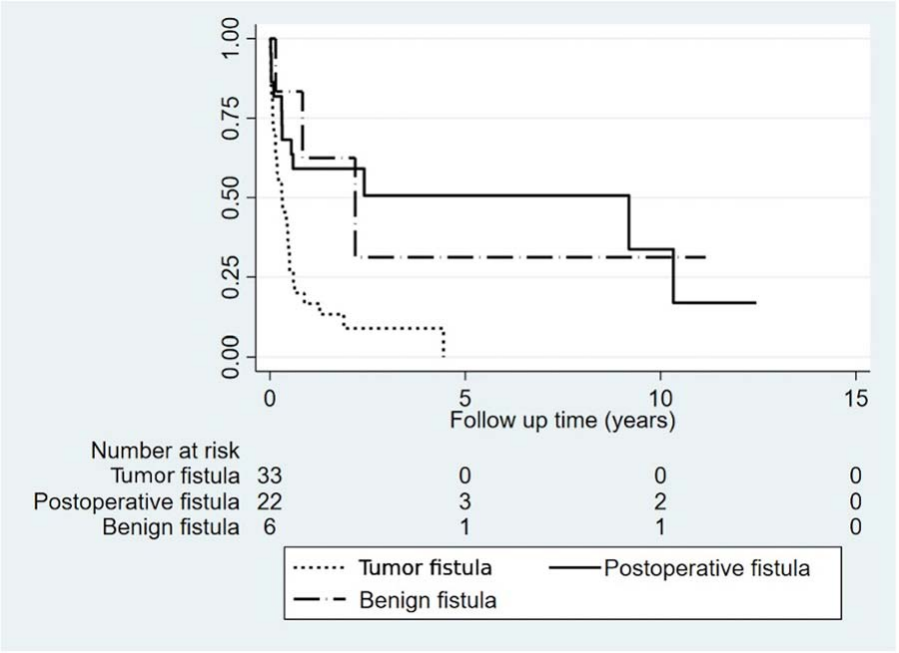
Survival stratified by aero-digestive fistula etiology.

For all patients, including those who received definitive surgery, and those who did not, 12 patients (19.7%) died within 30-days after diagnosis of fistula, nine (27.3%) in the tumor overgrowth ADF group, none in the benign fistula group and three in the post-esophagectomy group (13.6%). Twenty patients (32.8%) overall died within 90-days after diagnosis, 15 (45.5%) in the tumor overgrowth ADF group, one (16.7%1) in the benign ADF group and four (18.2%) in the post-esophagectomy group (Table 6 and Figs 2, 3).

## DISCUSSION

This unselected series presented 61 consecutive patients with ADF, most of whom were male, with acquired fistulas of various origins. Mortality in the cohort was high, especially in the tumor overgrowth-subgroup, overall lengths of hospital and ICU stays were long and most patients needed many re-interventions. The success rate of temporary sealing with SEMS, most often both in the alimentary tract and airway, was fairly high and can be considered feasible as bridge to definitive repair.

Aerodigestive fistulas are associated with high mortality and low quality of life for the patients. Because of the scant literature regarding ADFs the classification, management and treatment has progressed quite modestly in the last 20 years. A majority of the patients included in this study were previously diagnosed with a malignancy, and the largest group in this series comprised patients with a malignancy with tumor overgrowth and subsequent fistulation, in many cases after response to chemotherapy or chemoradiotherapy. ADF occurring as a post-operative complication after radical surgery with anastomotic leakage and conduit necrosis are also common causes of acquired ADF.^13^

Mortality and the need of intensive care are both high in patients with ADF. In this study, as well as in previous ones, this was due to complications such as aspiration, pneumonia and acute respiratory distress syndrome.^13^ To some extent the high mortality and morbidity may also be attributed to the study population’s high age and abundant co-morbidity, as we have shown this study.

In our institution, the treatment scheme has changed during the years of this study with a more active approach to definitive fistula repair and reconstruction of the gastrointestinal tract, when indicated. In our series, the postoperative 90-day mortality after definitive repair was 6.3%, which is low compared to some other series.^7^^,^^14^^,^^15^ This relatively low mortality may be attributed to our two-step approach with delayed elective surgery in well recovered patients, following the use of SEMS for temporary ADF sealing.^16^ However, as shown by Qureshi *et al*.^9^ SEMS can also be a cause of ADF, when used as a treatment for esophageal stenosis in esophageal carcinoma.

We do not have any reliable data on complications that can be clearly attributed to the double stenting because of difficulty in differentiating stent-caused complications from the clinical effects of the ADF in themselves. Generally the stents were well tolerated and suspicion of stent-related complications were rare. However, it’s clear that double tract stenting can cause some pressure necrosis of the esophageal and tracheo-broncheal wall, and increase the size of the fistula. In our cohort, this did not seem to affect the clinical outcomes in terms of temporary fistula sealing, and surgical repair was successful in a majority of cases, also with large fistulas.

A limitation of this study is its retrospective design. In addition, as this study includes patients from 2004–2022 and the technical quality of endoscopic, surgical, perioperative and intensive care may have changed during the study period, introducing additional heterogeneity into the data. Strengths of this study include the unselected consecutive case series and, for this relatively rare condition, a relatively large cohort.

In conclusion, this study demonstrates that ADF causes high morbidity and mortality in an upfront frail group of patients. ADF in adults is frequently caused by tumor overgrowth, ingestion of corrosive substances, as well as anastomotic leakage after esophagectomy. The management of these patient requires frequent multidisciplinary interventions and can be successful using a two-stage approach combining first temporary sealing with covered SEMS, followed by definitive surgery in an elective setting.

## Abbreviations

ADF, aerodigestive fistula; ASA, American Society of Anesthesiologists; ECOG, Eastern Cooperative Oncology Group.

## Notes

Financial support: None.
